# Anatomic femoral tunnel position in medial patellofemoral ligament reconstruction: anterior versus posterior

**DOI:** 10.1186/s12891-023-07069-3

**Published:** 2023-12-06

**Authors:** Kyoung Ho Yoon, Cheol Hee Park, Sung Hyun Hwang, Hyunjae Baek, Hee Sung Lee

**Affiliations:** 1 Department of Orthopaedic Surgery, Kyung Hee University Hospital, 23 Kyungheedae-ro, Dongdaemun-gu, Seoul, 02447 Republic of Korea; 2 Department of Orthopaedic Surgery, Pohang St. Mary’s Hospital, Pohang-si, Gyeongsangbuk-do Republic of Korea

**Keywords:** Medial patellofemoral ligament, Medial patellofemoral ligament reconstruction, Anatomic femoral tunnel position, Patellar instability

## Abstract

**Background:**

This study aimed to compare the clinical and radiological outcomes of medial patellofemoral ligament reconstruction (MPFLR) between anatomic femoral tunnel positions at anterior and posterior footprints.

**Methods:**

Fifty-seven patients who underwent MPFLR for patellofemoral instability with anterior or posterior femoral tunnels between 2014 and 2021 with at least 2 years of follow-up were retrospectively analyzed. Based on postoperative images, the femoral tunnel positions anterior to the line connecting the adductor tubercle and medial epicondyle were assigned to the anterior group, group A, and those posterior to the line to the posterior group, group P. Thirty-two patients were included in group A (mean age, 22.4 ± 8.8 years), and another 25 patients were included in group P (mean age, 21.1 ± 6.1 years). The International Knee Documentation Committee (IKDC) subjective score, Lysholm score, Tegner activity score, Kujala score, and complications were evaluated. Radiologically, the Caton–Deschamps index (CDI), patellar tilt angle, and patellofemoral osteoarthritis (PFOA) using the Kellgren–Lawrence (KL) scale were evaluated. The patellofemoral cartilage status according to the International Cartilage Repair Society (ICRS) grade, bone contusion, femoral tunnel enlargement, and MPFL graft signal intensity were also evaluated.

**Results:**

All clinical scores significantly improved in both groups (*p* < 0.01). No differences were noted between the two groups in terms of their preoperative demographic data, postoperative clinical scores (IKDC, Lysholm, Tegner, and Kujala), complications, or radiological findings (CDI, patellar tilt angle, PFOA, bone contusion, femoral tunnel enlargement, and graft signal intensity). The ICRS grade for the medial facet of the patella progressed in group A (30%, *p* = 0.02) but not in group P (18%, *p* = n.s.). Additionally, no significant differences were observed in the other compartments of the patellofemoral joint.

**Conclusions:**

The clinical outcomes were significantly improved in both groups; however, MPFLR with anterior femoral tunnel position had worse cartilage status on the medial facet of the patella than the posterior femoral tunnel position.

**Level of evidence:**

Level III.

## Introduction

Acute patella dislocation occurs primarily in young active patients [1]. The incidence of patellar dislocations has been reported to be as high as 23.2 per 100,000 person-years in the United States [2]. After a first dislocation, 17–42% are likely to experience further dislocations, which substantially increase the risk of developing subsequent patellofemoral instability, knee pain, and decreased knee function [3]. Medial patellofemoral ligament reconstruction (MPFLR) is the most commonly addressed surgical procedure to treat recurrent patellofemoral instability [4]. Several techniques have been described for femoral fixation with respect to tunnel position in MPFLR. Aframian et al. reported that MPFL inserts in a broad triangular space of the femur among the adductor tubercle (AT), medial epicondyle (ME), and gastrocnemius tubercle [5]. Some studies have suggested that the most optimal and isometric location for MPFLR is proximal and posterior to Schottle’s point, which is close to the AT [6–8]. Graft positioning anterior to the anatomic femoral MPFL attachment overconstrains the patellofemoral joint according to Kernkamp et al. [8]. A misplaced femoral attachment that is too anterior will result in excessive graft tension in deep flexion as the graft tries to stretch across the long anteroposterior dimension of the femoral condyle [9]. However, no correlation between MPFL femoral entry point positioning and subjective or objective outcome measures has been described in the literature [10–12]. The question on whether the femoral tunnel should be created anteriorly or posteriorly in MPFLR to achieve better outcomes remains to be answered. No clinical report has attempted to divide the femoral footprint of the MPFL with respect to the anterior or posterior anatomical location to date.

This study aimed to compare the clinical and radiological outcomes of MPFLR between anatomic femoral tunnel positions at anterior and posterior footprints. The hypothesis was that MPFLR in the anterior femoral tunnel position had worse clinical and radiological outcomes than that in the posterior femoral tunnel position.

## Materials and methods

### Patient selection and study design

**Fig. 1:**
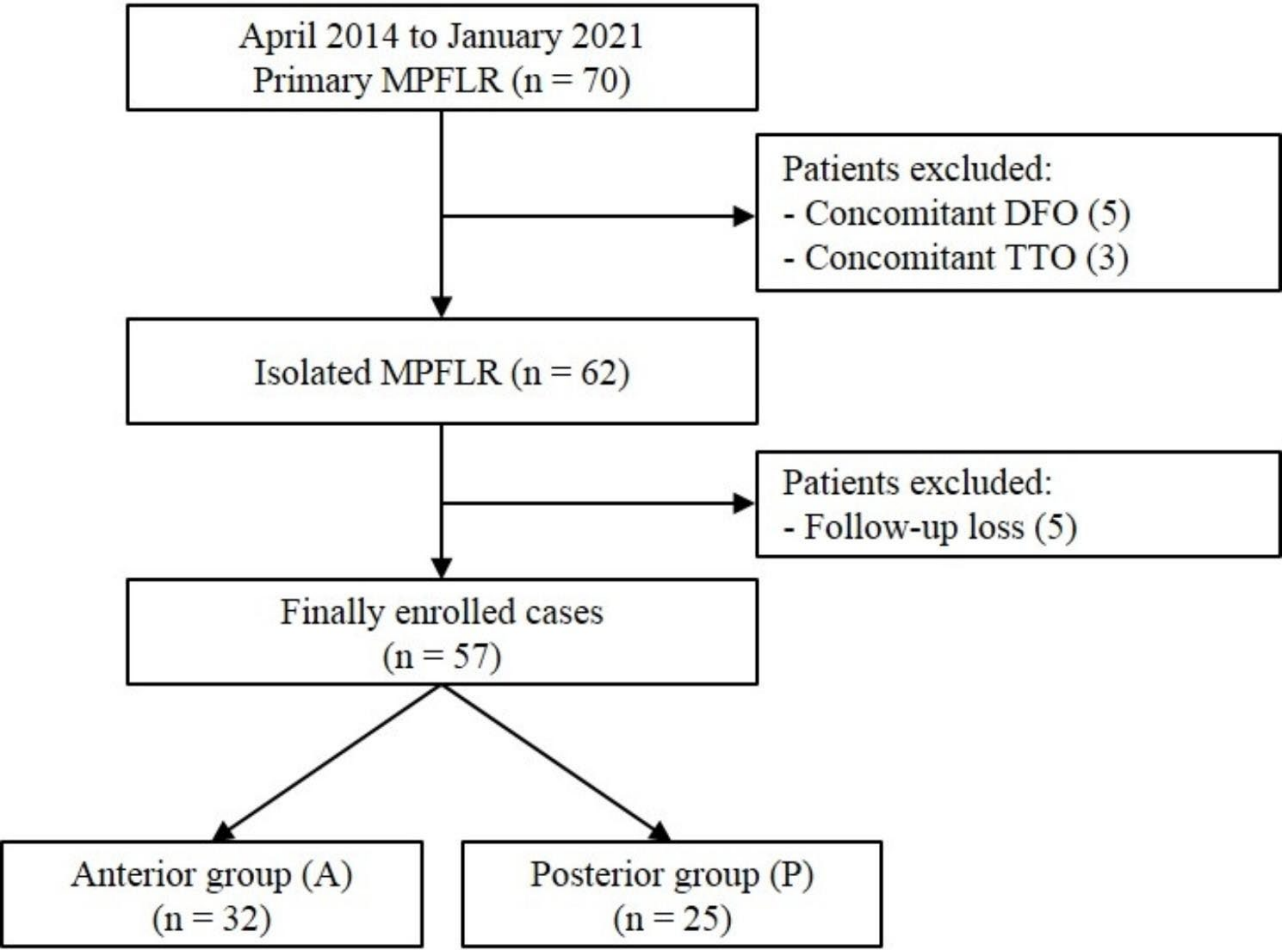
Flowchart of patient enrollment. MPFLR, medial patellofemoral ligament reconstruction; DFO, distal femoral osteotomy; TTO, tibial tuberosity osteotomy

The medical records and radiological data of the participants collected preoperatively and postoperatively were analyzed retrospectively after obtaining study approval from institutional review board. Patients who underwent isolated MPFLR for recurrent patellofemoral instability performed by a single surgeon between 2014 and 2021 were included. Patients with the underlying risk factors for isolated MPFLR failure were addressed with additional procedures when indicated, and excluded in this study: femoral anteversion angle greater than 30°, tibiofemoral valgus angle greater than 5°, tibial tuberosity–trochlear groove (TT–TG) distance greater than 20 mm, Dejour types C and D patella dysplasia [13, 14], and severe patellar alta based on radiography (Caton–Deschamps index [CDI] > 1.4) [15]. Other exclusion criteria were loss to follow-up, history of lower extremity injury or surgery, and absence of preoperative magnetic resonance imaging (MRI) or postoperative day 1 three-dimensional computed tomography (3D CT) scans. In addition, MRI scans were performed only for consenting patients 2 years after surgery. Fifty-seven patients with at least 2 years of follow-up were enrolled in the study (Fig. 1).

**Fig. 2:**
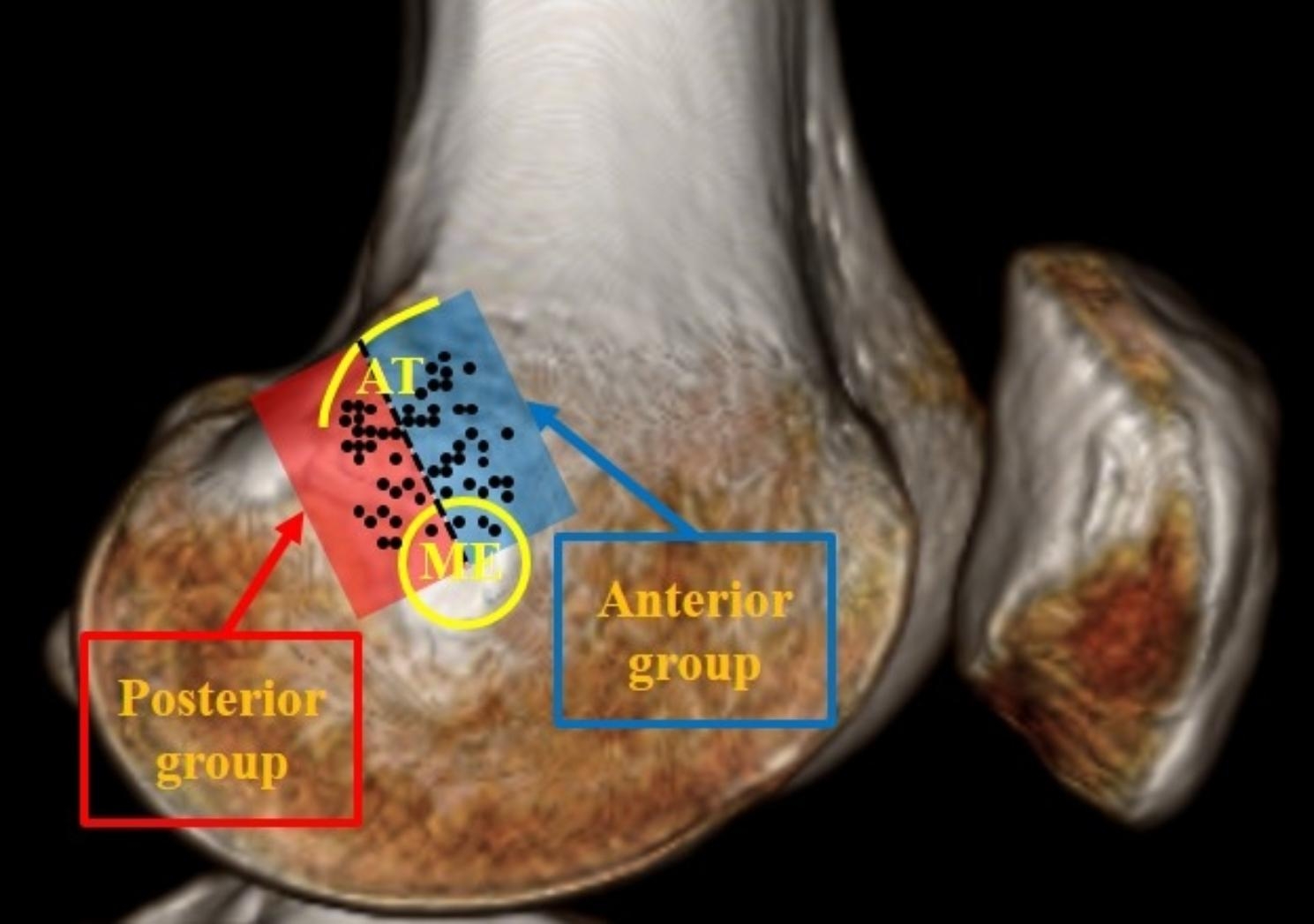
True medial image of 3D CT. The apex of the AT and the center of the ME were connected by a dotted line. The femoral tunnel positions (black dots) anterior to the dotted line (blue area) belong to the anterior group, and those posterior to the line (red area) belong to the posterior group. 3D CT, three-dimensional computed tomography; AT, adductor tubercle; ME, medial epicondyle

AT and ME were identified based on postoperative 3D CT. On the true medial image, the femoral tunnel positions anterior to the line connecting the AT and ME were assigned to the anterior group, and those posterior to the line to the posterior group (Fig. 2). Cases in which the femoral tunnel position occurred at a distance of less than 3 mm from the center line, which is half of the diameter of the tunnel drill size of 6 mm during surgery, were excluded. Patients who underwent MPFLR with anterior femoral tunnel position were classified as group A, and patients who underwent MPFLR with posterior femoral tunnel position were classified as group P.

### Patient demographics

Thirty-two patients (mean age, 22.4 ± 8.8 years) were assigned to group A and the remaining 25 patients (mean age, 21.1 ± 6.1 years) were assigned to group P. The preoperative demographic data did not differ between the groups (Table 1). Patella dysplasia was noted in 27 knees (84.4%) of group A (19 Dejour type A, 8 type B) and 21 knees (88%) of group P (14 type A, 8 type B).

**Table 1:** Preoperative demographic data^a^

	Group A	Group P	*p* Value
	(n = 32)	(n = 25)	
Age at surgery, year	22.4 ± 8.8	21.1 ± 6.1	n.s.
Male sex, n	13	11	n.s.
Injury side, right, n	19	9	n.s.
Body mass index, kg/m^2^	23.7 ± 4.1	22.5 ± 3.7	n.s.
Number of dislocations, n	1.1 ± 1.1	1.4 ± 1.1	n.s.
Interval^b^, months	7.0 ± 2.1	7.5 ± 2.0	n.s.
Follow-up period, months	32.8 ± 15.2	34.6 ± 13.0	n.s.
TT–TG distance, mm	10.6 ± 3.0	9.5 ± 3.2	n.s.
Patella dysplasia, n			n.s.
None	5	3	
Dejour type A	19	14	
Type B	8	8	
Type C or D	0	0	

^a^Values are presented as number or mean ± SD. Interval^b^, time interval from onset of instability to surgery; TT–TG, tibial tuberosity-trochlear groove; n.s., not significant

### Surgical technique and postoperative rehabilitation

Several landmarks were used for MPFLR, including the patella, vastus medialis, AT, and ME. A 3-cm longitudinal incision was made on the medial and proximal margins of the patella to which the vastus medialis tendon was attached. A 3.4-mm Healix Transtend BR™ (DePuy, Mitek, MA, USA) suture anchor was inserted into the patella at the upper third of the medial patellar border. A double-stranded tibialis anterior allograft with a 6-mm diameter looped end and two 4.5-mm diameter free ends was prepared. The two free ends of the graft were fixed to the patella using a suture anchor [16]. Another 3-cm longitudinal incision was made between the AT and ME. A femoral guide pin was inserted into the midpoint between the AT and ME after soft tissue dissection, direct visualization, and palpation of the femoral anatomic landmarks. Fluoroscopic guidance was not used during surgery. A 6-mm reaming was performed in the femoral tunnel [17]. Blunt dissection was performed carefully to make a tunnel in the second layer of medial soft tissue structures from the point of patellar insertion to the point of femoral insertion without damage to the capsule. Subsequently, a looped end of the graft was passed into the soft tissue tunnel and fixed with a 6-mm Biosure HA/PLLA (Smith & Nephew, Memphis, Tennessee, USA) in the femoral tunnel. Femoral fixation was performed with tension such that the lateral patellar edge was positioned in line with the lateral trochlear border at a knee flexion angle of 30°.

The rehabilitation program did not differ with respect to femoral tunnel position. Tolerable weight-bearing ambulation with a knee flexion angle of 30° cast was allowed during the first 6 weeks after MPFLR. The patients were encouraged to perform gradual range of motion exercises after 6 weeks. Full range of motion was achieved in all patients 3 months after MPFLR. Sports activities were allowed beginning at 6 months after surgery.

### Clinical evaluation

International Knee Documentation Committee (IKDC) subjective score [18], Lysholm score [19], Tegner activity score [20], Kujala score [21], and complications (redislocation, patellar fracture, infection, or stiffness) were assessed preoperatively and 2 years postoperatively for all patients who underwent MPFLR. Kujala improvement was considered the primary outcome [22, 23]. Kujala improvement was considered the average change in the Kujala score measured as the difference between the postoperative and preoperative Kujala scores.

### Radiological evaluation

Radiologic evaluations including CDI, patellar tilt angle, and patellofemoral osteoarthritis (PFOA) using the Kellgren–Lawrence (KL) scale on plain radiographs were conducted preoperatively and 2 years after surgery. Data were analyzed for intra-group and inter-group comparisons. Irrespective of symptoms, only patients who consented to MRI examination (3.0-T Achieva; Philips Medical Systems, Andover, MA, USA) using a T2-weight turbospin-echo sequence (matrix 320, time repetition [TR] 3774 ms, time echo [TE] 100 ms, 2.5 mm thickness) in sagittal and coronal planes, and a proton density turbospin-echo sequence (matrix 340, TR 4350 ms, TE 30 ms, 3 mm thickness) in three planes (axial, sagittal, and coronal) one year after surgery were performed. The patellofemoral cartilage status using the International Cartilage Repair Society (ICRS) grade compared with that of preoperative MRI, bone contusion, femoral tunnel enlargement, and MPFL graft signal intensity were assessed. The patellofemoral cartilage status was evaluated by dividing it into four compartments (medial and lateral facets of the patella and trochlea) to exclude already fibrillated or eroded cartilage due to instability episodes [24, 25]. Progression of PFOA or ICRS grade was defined as increase in postoperative KL or ICRS grade by ≥ 1 grade than in the preoperative state. Bone contusion was evaluated according to the aforementioned four compartments.

All parameters were measured using a picture archiving and communication system (Pi-View STAR software; INFINITT, Seoul, South Korea). To minimize observation bias, two orthopedic surgeons who did not participate in the surgeries performed all the radiographic measurements and assessment was repeated 6 weeks later by the senior surgeon. The interobserver and intraobserver kappa values were 0.89 and 0.91, respectively.

### Measurement of cross-sectional area (CSA)

**Fig. 3:**
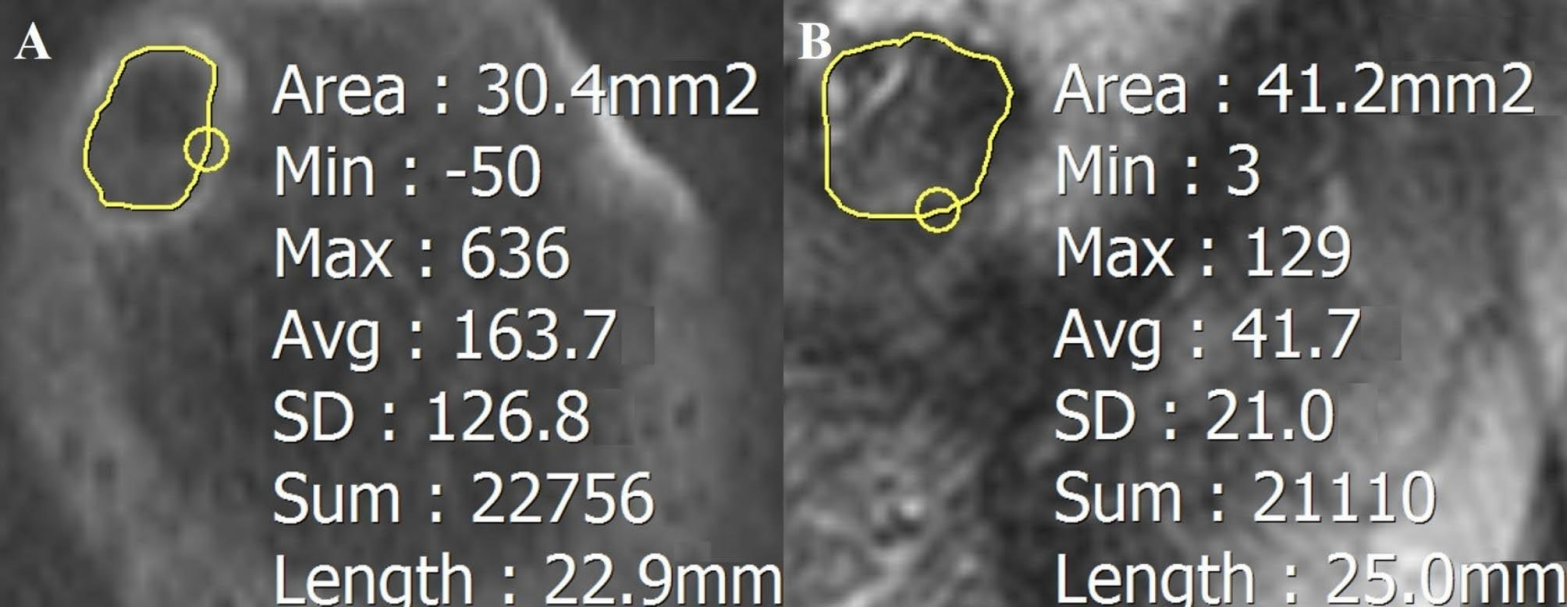
Measurement of the CSA. The lining along the aperture boundary was recorded for the CSA of the femoral tunnel using the freehand technique. (**A**) CSA on CT at 1 day postoperatively, (**B**) CSA on MRI at 2 years postoperatively. CSA, cross-sectional area; CT, computed tomography; MRI, magnetic resonance imaging

A 64-slice CT (General Electric, Boston, MA, USA) of the affected knee was obtained 1 day postoperatively for all patients and MRI at 2 years postoperatively for consenting patients. The CSA of the femoral tunnel was measured on a plane perpendicular to the long axis of the femoral tunnel. The most medial plane in which the entire tunnel wall was surrounded by bone is defined as the aperture as previously described by Kita et al. [9]. The lining along the aperture boundary was recorded for the CSA of the femoral tunnel using the freehand technique (Fig. 3). The CSAs of the femoral tunnel obtained from the postoperative 1-day CT and 2-year MRI scans were compared between the two groups. The CSAs of the aperture 1 day after surgery as the reference value were compared with those at 2 years to evaluate femoral tunnel enlargement.

### Measurement of graft signal intensity on MRI

Graft signal intensity was classified at three sites of MPFL grafts (patellar insertion, mid-substance, and femoral insertion) based on T2-weighted images (low intensity was the same as that of the patellar tendon, intermediate intensity was the same as that of the gastrocnemius muscle, and high intensity was greater than intermediate intensity) according to the protocol of Figueroa et al. [26, 27].

### Statistical analysis

All statistical analyses were performed using SPSS version 22.0 (IBM Inc., Chicago, IL, USA). Pearson’s chi-squared test or Fisher’s exact test was performed, and expressed as frequencies and percentages for categorical data. The Kolmogorov–Smirnov test was performed to test the normality for continuous data, and the results are presented as the mean and standard deviation (SD). An independent t-test was performed to compare normally distributed continuous variables, whereas the Mann–Whitney U test was performed to compare non-normally distributed continuous variables. The paired-sample t-test was used for normally distributed data for preoperative and postoperative comparisons of dependent variables, and the Wilcoxon signed-rank test was used for non-normally distributed data. *P* Values < 0.05 were considered statistically significant. Sample size calculation was performed according to a previous study based on a significant difference in the mean Kujala score (8 ± 8 points) [28]. At least 34 patients (17 individuals in each group) were required to detect significant changes with 80% power and 95% confidence.

## Results

### Clinical outcomes

All clinical scores (IKDC, Lysholm, Tegner, and Kujala) significantly improved in both groups, whereas none of the scores showed differences between the groups at the 2-year follow-up visit. Additionally, no complications were observed in either group at a follow-up of at least 2 years (Table 2).

**Table 2:** Clinical outcomes after the 2-year follow-up period^a^

	Group A	Group P	*p* Value
	(n = 32)	(n = 25)	
IKDC subjective score			
Preoperative	40.3 ± 6.5	40.6 ± 9.1	n.s.
Postoperative	74.1 ± 15.4	71.4 ± 13.8	n.s.
*p* Value	< 0.01	< 0.01	
Lysholm score			
Preoperative	40.3 ± 5.8	42.4 ± 10.4	n.s.
Postoperative	76.8 ± 13.9	81.3 ± 11.0	n.s.
*p* Value	< 0.01	< 0.01	
Tegner activity scale, median (range)			
Preoperative	3 (1–5)	3 (1–6)	n.s.
Postoperative	5 (3–7)	6 (3–8)	n.s.
*p* Value	< 0.01	< 0.01	
Kujala score			
Preoperative	42.7 ± 5.9	40.8 ± 3.8	n.s.
Postoperative	79.5 ± 8.4	79.0 ± 4.9	n.s.
*p* Value	< 0.01	< 0.01	
Complications, n	0	0	NA

^a^Values are presented as number or mean ± SD. IKDC, International Knee Documentation Committee; n.s., not significant

### Radiologic outcomes

The patellar height did not differ between the two groups. The patellar tilt improved; however, was not different between the two groups. The progression of PFOA did not differ between the two groups (Table 3).

**Table 3:** Radiologic outcomes after the 2-year follow-up period^a^

	Group A	Group P	*p* Value
	(n = 32)	(n = 25)	
CDI			
Preoperative	1.1 ± 0.3	1.1 ± 0.3	n.s.
Postoperative	1.0 ± 0.4	1.0 ± 0.3	n.s.
*p* Value	n.s.	n.s.	
Patellar tilt angle, °			
Preoperative	17.4 ± 5.1	18.9 ± 7.5	n.s.
Postoperative	11.7 ± 4.1	11.8 ± 3.6	n.s.
*p* Value	< 0.01	< 0.01	
PFOA^b^, n			
Preoperative			
Gr 0 / 1 / 2 / 3 or 4	27 / 5 / 0 / 0	19 / 5 / 1 / 0	n.s.
Postoperative			
Gr 0 / 1 / 2 / 3 or 4	25 / 6 / 1 / 0	16 / 8 / 1 / 0	n.s.
*p* Value	n.s.	n.s.	
PFOA progression, n	3	3	n.s.

^a^Values are presented as number or mean ± SD. ^b^Values are evaluated using the Kellgren-Lawrence scale. CDI, Caton–Deschamps index; PFOA, patellofemoral osteoarthritis; n.s., not significant

Twenty patients in group A and 17 patients in group P agreed to undergo MRI scans at 2-year follow-up. The remaining patients did not have available postoperative MRI. The ICRS grade for the medial facet of the patella progressed in group A (6/20, *p* = 0.02) but not in group P (3/17, *p* = n.s.). However, no significant differences were observed in the other compartments of the patellofemoral joint. Additionally, no differences were noted between the two groups with regard to bone contusion of the patellofemoral compartment, femoral tunnel enlargement, and MPFL graft signal intensity (Table 4).

**Table 4:** Magnetic resonance imaging outcomes after the 2-year follow-up period^a^

	Group A	Group P	*p* Value
	(n = 20)	(n = 17)	
Cartilage status^b^, n			
Medial facet of the patella			
Preoperative Gr 0 / 1 / 2 / 3 / 4	0 / 6 / 9 / 4 / 1	0 / 6 / 6 / 5 / 0	n.s.
Postoperative Gr 0 / 1 / 2 / 3 / 4	0 / 3 / 10 / 5 / 2	0 / 5 / 6 / 6 / 0	n.s.
*p* Value	0.02	n.s.	
Lateral facet of the patella			
Preoperative Gr 0 / 1 / 2 / 3 / 4	0 / 10 / 7 / 3 / 0	0 / 12 / 5 / 0 / 0	n.s.
Postoperative Gr 0 / 1 / 2 / 3 / 4	0 / 9 / 8 / 3 / 0	0 / 11 / 6 / 0 / 0	n.s.
*p* Value	n.s.	n.s.	
Medial facet of the trochlea			
Preoperative Gr 0 / 1 / 2 / 3 / 4	0 / 17 / 2 / 1 / 0	0 / 15 / 1 / 1 / 0	n.s.
Postoperative Gr 0 / 1 / 2 / 3 / 4	0 / 16 / 3 / 1 / 0	0 / 15 / 1 / 1 / 0	n.s.
*p* Value	n.s.	NA	
Lateral facet of the trochlea			
Preoperative Gr 0 / 1 / 2 / 3 / 4	0 / 13 / 6 / 1 / 0	0 / 12 / 3 / 2 / 0	n.s.
Postoperative Gr 0 / 1 / 2 / 3 / 4	0 / 13 / 6 / 1 / 0	0 / 12 / 3 / 2 / 0	n.s.
*p* Value	NA	NA	
ICRS grade progression, n			
Medial facet of the patella	6	2	n.s.
Lateral facet of the patella	1	1	n.s.
Medial facet of the trochlea	1	0	n.s.
Lateral facet of the trochlea	0	0	NA
Bone contusion, n			
Medial facet of the patella	8	5	n.s.
Lateral facet of the patella	1	0	n.s.
Medial facet of the trochlea	4	2	n.s.
Lateral facet of the trochlea	1	0	n.s.
Cross-sectional area of the femoral tunnel, mm^2^			
1: Postoperative, 1 day, CT	30.4 ± 1.3	30.6 ± 1.1	n.s.
2: Postoperative, 2 years, MRI	42.6 ± 6.0	43.7 ± 4.4	n.s.
Femoral tunnel enlargement (%)			
1 vs 2	50.6 ± 21.2	52.7 ± 15.5	n.s.
*p* Value	< 0.01	< 0.01	
Graft signal intensity, n			
Patellar insertion Low / Intermediate / High	8/11/2001	7/7/2003	n.s.
Mid-substance Low / Intermediate / High	14 / 6 / 0	10/7/2000	n.s.
Femur insertion Low / Intermediate / High	13 / 7 / 0	10/6/2001	n.s.

^a^Values are presented as number or mean ± SD, ^b^Values are evaluated using the ICRS grade. ICRS, International Cartilage Repair Society; CT, computed tomography; MRI, magnetic resonance imaging; n.s., not significant

## Discussion

The most important finding of this study was that the clinical and radiological outcomes between groups A and P were comparable, even though group A had worse cartilage status on the medial facet of the patella than group P on the 2-year follow-up MRI. The results of this study may help guide surgical planning, maximize functional recovery, and improve prognosis in patients with recurrent patellofemoral instability.

Several studies have reported excellent clinical and radiological outcomes after MPFLR [14, 29, 30]. When patients with high-grade patella dysplasia (Dejour type C or D) were excluded, anatomically placed femoral tunnels demonstrated significantly better clinical scores, 83% of patients were either very satisfied or satisfied with the outcome of their surgery, and 56% returned to sport postoperatively [14]. Complications following MPFLR ranged from 0 to 32.3% [31]. The large cohort with a mean follow-up of 4.9 years concluded a significant correction in patellar tilt is correlated with good clinical results [29]. Similar to previous studies, the current study also demonstrated that MPFLR in both groups showed significant overall improvement in clinical outcomes and patellar tilt angle.

Shatrov el at [32]. reported some degenerative changes will develop in one-third of the patients at 12.3 years after MPFLR. Recent studies have reported that the femoral tunnel position anterior to the anatomic origin could increase medial patellofemoral compartment pressures resulting from overtensioned MPFL grafts and potentially lead to osteoarthritis [8, 33–35]. The current study was consistent with several reports in that no significant progression of PFOA was noted after MPFLR [16, 30, 36]. Interestingly, unlike the results of the radiographic evaluation, MRI evaluation of cartilage status showed significant progression of the ICRS grade for the medial facet of the patella in group A (*p* = 0.02), but not in group P (*p* = n.s.). The latter result implies that anterior femoral attachment may increase medial pressure. However, the medial trochlear cartilage (rather than the medial patellar cartilage) and bone contusion did not differ between the groups. The underlying mechanism for this finding is not fully understood. First, this may be a result of the small number of samples due to the limited number of postoperative MRI scans. Second, previous studies considered femoral tunnel malposition when the center is more than 10 mm away from Schottle’s point according to Servien et al. [10]. In the present study, comparison of anterior and posterior tunnel positions within the range of anatomic footprints between AT and ME may also affect the results.

Six to 12 months after anterior cruciate ligament reconstruction is the peak of femoral tunnel enlargement according to Weber et al. [37], and performing MRI is appropriate at 2 years after surgery. Kita et al. [9] reported a 41.1% enlargement of the femoral tunnel on CT 1 year after MPFLR. Femoral tunnel enlargement (50.6% in group A, *p* < 0.01; 52.7% in group P, *p* < 0.01) was observed at 2 years after MPFLR. However, the tunnel enlargement was not significantly different between the groups. Fixation of the femoral tunnel within the anatomic footprints between AT and ME may affect the results.

This study has several limitations. First, it was retrospective. Therefore, the study was non-randomized and affected by selection bias. A prospective randomized study with a more elaborate design would have provided more reliable data, although no differences were noted in patient demographics or preoperative status between groups A and P. Second, the number of enrolled patients was small, which could have caused type 2 errors. Some femoral tunnels were located near the central position; however, we could not categorize them into an additional group given the limitations of the statistical techniques and small sample sizes. If the number of patients was enough, some differences could have been observed through dividing the cases into anterior, posterior, and central groups for the femoral tunnel positions. Further studies with larger sample sizes are necessary to obtain more precise conclusions. Third, it is a short-term result overall, and since the MRI scan results of some patients were analyzed 2 years after surgery, it might not be sufficient to confirm complications such as PFOA progression. Therefore, mid- to long-term follow-up studies are warranted in the future.

## Conclusions

The clinical outcomes were significantly improved in both groups; however, MPFLR with anterior femoral tunnel position had worse cartilage status on the medial facet of the patella than the posterior femoral tunnel position.

## Data Availability

The data-sets used and/or analysed during the current study available from the corresponding author on reasonable request.

## List of abbreviations

3D CT: Three-dimensional computed tomography
AT: Adductor tubercle
CDI: Caton–Deschamps index
CSA: Cross-sectional area
DFO: Distal femoral osteotomy
ICRS: International Cartilage Repair Society
IKDC: International Knee Documentation Committee
KL: Kellgren–Lawrence
ME: Medial epicondyle
MPFL: Medial patellofemoral ligament
MPFLR: Medial patellofemoral ligament reconstruction
MRI: Magnetic resonance imaging
PFOA: Patellofemoral osteoarthritis
SD: Standard deviation
TT–TG: Tibial tuberosity–trochlear groove
TTO: Tibial tuberosity osteotomy
